# Chronic fluoxetine treatment in middle-aged rats induces changes in the expression of plasticity-related molecules and in neurogenesis

**DOI:** 10.1186/1471-2202-13-5

**Published:** 2012-01-05

**Authors:** Ramon Guirado, David Sanchez-Matarredona, Emilo Varea, Carlos Crespo, José Miguel Blasco-Ibáñez, Juan Nacher

**Affiliations:** 1 Neurobiology Unit and Program in Basic and Applied Neurosciences, Cell Biology Dpt., Universitat de València, Spain; 2 INCLIVA: Fundación Hospital Clínico Universitario de Valencia, València, Spain

## Abstract

**Background:**

Antidepressants promote neuronal structural plasticity in young-adult rodents, but little is known of their effects on older animals. The polysialylated form of the neural cell adhesion molecule (PSA-NCAM) may mediate these structural changes through its anti-adhesive properties. PSA-NCAM is expressed in immature neurons and in a subpopulation of mature interneurons and its expression is modulated by antidepressants in the telencephalon of young-adult rodents.

**Results:**

We have analyzed the effects of 14 days of fluoxetine treatment on the density of puncta expressing PSA-NCAM and different presynaptic markers in the medial prefrontal cortex, hippocampus and amygdala of middle-aged (8 months old) rats. The density of puncta expressing PSA-NCAM increased in the dorsal cingulate cortex, as well as in different hippocampal and amygdaloid regions. In these later regions there were also increases in the density of puncta expressing glutamic acid decarboxylase 65/67 (GAD6), synaptophysin (SYN), PSA-NCAM/SYN and PSA-NCAM/GAD6, but a decrease of those expressing vesicular glutamate transporter 1 (VGluT1). Since there is controversy on the effects of antidepressants on neurogenesis during aging, we analyzed the number of proliferating cells expressing Ki67 and that of immature neurons expressing doublecortin or PSA-NCAM. No significant changes were found in the subgranular zone, but the number of proliferating cells decreased in the subventricular zone.

**Conclusions:**

These results indicate that the effects of fluoxetine in middle-aged rats are different to those previously described in young-adult animals, being more restricted in the mPFC and even following an opposite direction in the amygdala or the subventricular zone.

## Background

Recent hypotheses support the idea that abnormalities in neuronal structural plasticity may underlie the etiopathogenesis of major depression [1,2]. Accordingly, it has been shown that patients and animal models of this disorder show changes in the volume of certain cerebral regions, such as the hippocampus, the medial prefrontal cortex or the amygdala, which are related to the reorganization of neuronal structure and may affect their connectivity [3,4]. However, these structural changes are not limited to neuronal remodeling, they may also affect the production of new neurons, specially in the adult hippocampus [5-8]. Interestingly, antidepressant treatment is able to revert or block this plasticity in experimental animals and humans [9-13]. In fact, different lines of evidence indicate that both neuronal remodeling [14-17] and adult neurogenesis [12,18] may play an important role in the way of action of antidepressant drugs.

The regulation of the expression of cell adhesion molecules is critical for the neuronal structural remodeling induced by aversive experiences or by antidepressant treatments [19]. Previous results from our laboratory have shown that chronic treatment with the serotonin (5HT) reuptake inhibitor fluoxetine, a commonly used antidepressant, influences the expression of the polysialylated form of the neural cell adhesion molecule (PSA-NCAM) in different regions of the CNS of young-adult animals [20,21]. Similar results have been obtained with another antidepressant, imipramine [17]. PSA-NCAM, due to its anti-adhesive properties, creates a steric impediment for cell adhesion and, consequently, promotes structural plasticity [22]. In fact, the changes in PSA-NCAM expression induced by chronic fluoxetine treatment are accompanied by parallel changes in the expression of the synaptic protein synaptophysin (SYN), suggesting the occurrence of synaptic remodeling [21]. PSA-NCAM expression is abundant during development and, although it decreases markedly during adulthood, it is still detectable in many cerebral regions, such as the medial prefrontal cortex (mPFC) [23,24], amygdala [25] and hippocampus [26], which are known to be involved in major depression. In these regions, PSA-NCAM is expressed in immature neu-rons, such as those in the hippocampal subgranular zone (SGZ) [27] and the subventricular zone (SVZ) [28], but it is also expressed in a subpopulation of mature interneurons [29,24], which have reduced syn-aptic input and morphological features compared with other interneurons lacking PSA-NCAM [30]. Pre-vious work in our laboratory demonstrated that 5HT, acting via 5HT3 receptors is able to regulate PSA-NCAM expression in the mPFC of adult rats [20].

The effects of antidepressants on neuronal plasticity are not restricted to structural remodeling of neurons and their connections, they may also influence the generation and incorporation of new neurons in the adult CNS. Chronic antidepressant treatments increase neurogenesis in the SGZ of the dentate gyrus and the SVZ of young-adult rodents [31,32], and this increase appears to be required at least for part of the behavioral improvement observed in the treated animals [18]. However, other studies are in disagreement with these findings and have found that antidepressants do not produce an increase in neurogenesis in the SGZ [33,34].

Despite all these interesting findings regarding the effects of antidepressants on neuronal plasticity, it has to be noted that most of the experiments have been performed using young-adult rodents, usually 2-3 months old. Consequently, there is controversy on whether antidepressants, and specifically 5HT reuptake inhibitors, exhibit the same efficacy within different age groups. This is particularly interesting because aging influences both neurogenesis [35] and PSA-NCAM expression [36]. Studies on the effects of antidepressant treatment using older animals are still scarce: Only a few works have studied neurogenesis in the SGZ [32,37], but not in the SVZ and, to our knowledge, none has studied other types of neuronal structural plasticity.

We have analyzed the effects of 14 days of chronic fluoxetine treatment on middle-aged (8 month old) rat brains, studying the expression of PSA-NCAM and that of different presynaptic proteins in regions known to be specially affected in patients and in animal models of major depression, as well as by antidepressant treatment, such as the mPFC, the amygdala, and the hippocampus [38-42]. We have studied the expression of SYN a general synaptic marker, glutamic acid decarboxylase 65/67 (GAD6), a marker for inhibitory synapses and the vesicular glutamate transporter 1 (VGluT1), a marker of excitatory synapses. To study the effects of fluoxetine on adult neurogenesis, we have analyzed the numbers of im-mature cells, using immunohistochemistry for doublecortin (DCX) and PSA-NCAM, and those of prolife-rating cells with Ki67 in the two neurogenic niches of the adult brain: the SGZ and the SVZ.

## Methods

### Animal treatments

Fourteen adult male Wistar rats (8 months old) were used in this experiment. All the rats were maintained in standard conditions of light (12 hour cycles) and temperature, with no limit in the access to food or water. All animal experimentation was conducted in accordance with the Directive 2010/63/EU of the European Parliament and of the Council of 22 September 2010 on the protection of animals used for scientific purposes and was approved by the Committee on Bioethics of the Universitat de València. Every effort was made to minimize the number of animals used and their suffering. Rats were chronically injected intraperitoneally either with the antidepressant fluoxetine (n = 7, 10 mg/kg), or with saline solution (n = 7), during 14 days (once daily at 10.00 am). Previous studies have shown that this dose produces behavioral changes and increases in the expression of plasticity-related molecules [21,58]. The day after these treatments, rats were perfused transcardially under deep chloral hydrate anesthesia (chloral hydrate 4%, 1 ml/100 gr), first with saline and then with 4% paraformaldehyde in so-dium phosphate buffer (PB 0.1 M, pH 7.4). After perfusion, the brains were extracted and cryoprotected with 30% sucrose in PB. Brains were cut into 50 μm thick sections with a freezing sliding microtome, collected in 10 subseries and stored at - 20°C in 30% glycerol, 30% ethylene glycol in PB 0.1 M until used.

### Immunohistochemistry

Tissue was processed free-floating as follows: Briefly, sections were incubated for 1 min in an antigen unmasking solution (0.01 M citrate buffer, pH 6) at 100°C. After cooling down the sections to room temperature, the endogenous peroxidase was blocked with 10 min incubation in a solution of 3% H_2_O_2 _in phosphate buffered saline (PBS). Afterwards, slices were incubated in 10% normal donkey serum (NDS) (Abcys SA), 0.2% Triton-X100 (Sigma) in PBS with for 1 hour; then, they were incubated overnight at room temperature with goat polyclonal anti-doublecortin (DCX) (C-18; 1:250; Santa Cruz Biotechnology, Inc.), mouse monoclonal anti-PSA-NCAM IgM (1:1400; Abcys SA) or mouse monoclonal anti-Ki67 IgG (1:2000; Novocastra). After washing, sections were incubated for 30 min with biotinylated donkey anti-goat IgG, donkey anti-mouse IgM or donkey anti-mouse IgG (1:200; Jackson Immunoresearch) antibodies, followed by an avidin-biotin-peroxidase complex (ABC, Vector Laboratories) for 30 min in PBS. Color development was achieved by incubating with 0,5% 3,3' diaminobenzidine tetrahydrochloride (DAB, Sigma) and H2O2 for 4 min. All the sections passed through all procedures simultaneously to minimize any difference from immunohistochemical staining itself.

For double fluorescence immunohistochemistry, slices were incubated overnight with rabbit polyclonal anti-synaptophysin (SYN) IgG (1:1000; Chemicon Int. Inc.) and mouse monoclonal anti-PSA-NCAM IgM (1:1400; Abcys SA). Then, were incubated for 1 hour with donkey anti-rabbit IgG Dylight 488 and donkey anti-mouse IgM Dylight 555 (1:200; Jackson Immunoresearch).

For triple immunohistochemistry, the procedure was similar but using a combination of anti-PSA-NCAM, anti-glutamic acid decarboxylase 65/67 (GAD6; 1:500; Developmental Studies Hybridoma Bank) and anti-vesicular glutamate transporter (VGluT1; 1:2000; Chemicon Int. Inc.) antibodies. In this case the following secondary antibodies were used: anti-mouse IgM Dylight 555, anti-mouse IgG subtype 1 specific Dylight 488 and anti-guinea pig IgG Dylight 647 respectively.

### Quantitative analysis

All the slides containing sections destined to quantitative analysis were coded and their code was not broken until the analyses were finished.

The volume of the mPFC, the hippocampus and the lateral and basolateral region of the amygdala was estimated using Volumest, an ImageJ plugin for volume estimation using an stereological method. For this analysis we used the tissue processed with PSA-NCAM immunohistochemistry and developed with DAB was used.

The images used for the analysis of neuropil puncta were obtained with a confocal microscope (Leica TCS SPE) and parallel subseries of Nissl stained sections were used as guides to help locate the regions of interest. We analyzed layer V of two different regions of the medial prefrontal cortex (mPFC): the prelimbic cortex (PrL) in **a single **section corresponding to Bregma +2.20 mm and **another one of **the dorsal cingulate cortex (Cg2) (Bregma +1 mm). In the amygdala, five nuclei were analyzed **in one section **(Bregma -3.30 mm): basomedial, lateral, medial, central and basolateral. Different regions and strata of the hippocampus were also analyzed in **another **section (Bregma -4.30 mm): the molecular layer of the dentate gyrus, the strata lacunosum-moleculare, radiatum and oriens of CA1 and the stratum lucidum of CA3. Confocal z-stacks covering the whole depth of the sections were taken with 1 μm step size and only subsets of confocal planes with the optimal penetration level for each antibody were selected. On these planes, small regions of the neuropil (505 μm^2^) were selected for analysis, in order to avoid blood vessels and cell somata.

Images were processed using ImageJ software as follows: the background was substracted with rolling value of 50, converted to 8-bit deep images and binarized using a determined threshold value. This value depended on the marker and the area analyzed and was kept the same for all images with the same marker and area. Then, the images were processed with a blur filter to reduce noise and separate closely apposed puncta. Finally, the number of the resulting dots per region was counted, as well as the colocalization between PSA-NCAM and the different pre-synaptic markers (Additional File 1, Figure S1). **In addition, we also analyzed the size of the dots and the total surface covered by the puncta after binarization**. Means were determined for each animal group and the data were subjected to two-way ANOVAs with repeated measures followed by Bonferroni *post hoc *test.

The density of DCX, PSA-NCAM and Ki67 expressing cells in the SGZ of the dentate gyrus was estimated as described before [25]. Briefly, sections were selected by a 1:10 fractionator sampling covering the whole rostral to caudal extension of the dentate gyrus and on each section all labeled cells within the region of interest were counted. Cell somata were identified and counted with a 40 × objective using an Olympus CX41 microscope. The volume of the different areas analyzed was determined for each animal using the Cavalieri's method. Student's t-test was performed for statistical analysis.

In the SVZ, a single section between Bregma +0.48 and +0.7 mm, was analyzed for DCX, PSA-NCAM and Ki67 immunohistochemistry. The number of immunoreactive cells was estimated following a modification of the method described by Hansson *et al*. [43]. Ki67 expressing nuclei were counted using automatic counting software as described above for immunoreactive puncta, analyzing an area of 100 μm^2 ^and then expressed as the number of immunoreactive positive cells per mm^2^. Densities of DCX and PSA-NCAM expressing cells were too high for a correct individual cell count. Therefore, we performed densitometry of small areas within the SVZ, using a 40 × magnification, as described before [20].

## Results

### Weight of the animals and volume of the different structures analyzed

**Figure 1:**
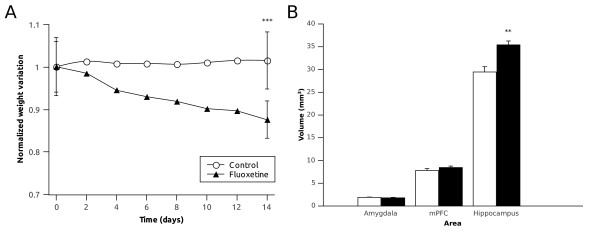
**Graphs showing (A) the body weight change through the experiment for the control and fluoxetine group and (B) the effect of the chronic fluoxetine treatment on the volume of the different structures studied**. Statistically significant (* P < 0.05, ** P < 0.01, *** P < 0.001) Student t-test.

In concordance with previous studies [44,45], chronic fluoxetine treatment produced a significant diminution of weight gain (p < 0.001) after 14 days, when comparing the relation final weight/initial weight between the treated animals (0.877 ± 0.017) and the controls (1.017 ± 0.009) (Figure 1).

**We also analyzed the effects of chronic fluoxetine treatment on the volume of the different cerebral structures studied. We did not observe changes in the volume of the mPFC or the amygdala, but we found an increase in the volume of the hippocampus of the animals treated with fluoxetine (p = 0.001, Figure **1**).**

### Expression of plasticity-related Molecules

#### Medial prefrontal cortex

**Figure 2:**
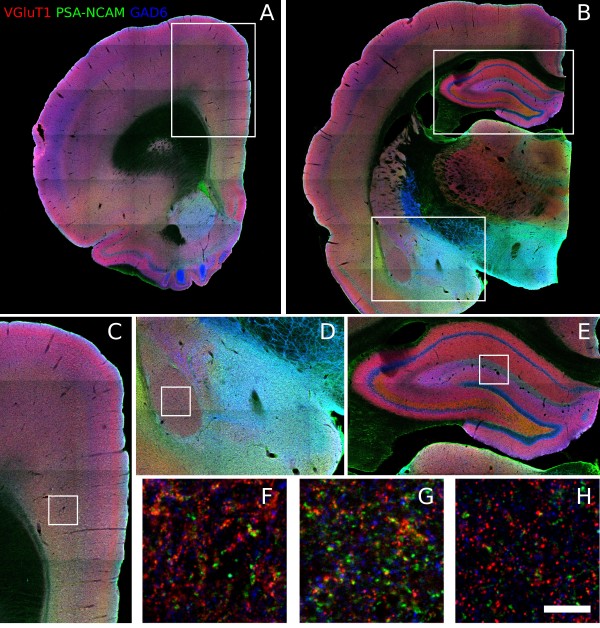
**Confocal images showing the expression of PSA-NCAM, VGluT1 and GAD6 in the different regions of the hippocampus (B, E and H), the amygdala (B, D and G) and the mPFC (A, C and F)**. Scale bar = 200 μm in A and B, 100 μm in C, D and E and 1 μm in F, G and H.

The expression pattern of PSA-NCAM in the mPFC was similar to that described previously [23]: All regions within the mPFC showed a moderate intensity of staining in layer I, nearly lack of staining in layer II, weak staining in layer III and intense staining in layers V-VI (Figure 2).

**Figure 3:**
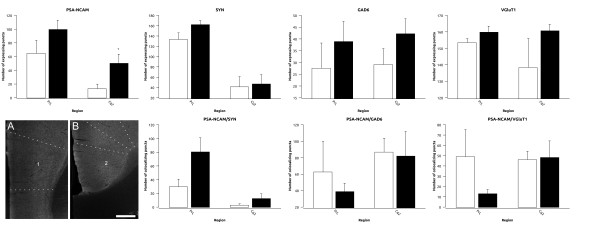
**Graphs representing the expression of puncta for the different markers and its colocalization in the prelimbic and cingulate cortex of the medial prefrontal cortex**. White bars represent control animals and black bars represent fluoxetine treated animals respectively. A and B are focal planes showing the expression of PSA-NCAM in (1) the prelimbic and (2) the cingulate cortex. Scale bar: 200 μm.

In this cortical region, there was an increase in the density of PSA-NCAM expressing puncta in the fluoxetine treated animals in the dorsal cingulate cortex (Cg2) (p = 0.043), but not in the prelimbic cortex (PrL) (Figure 3 and Additional File 2, Table S1). No differences were found in the density of puncta expressing synaptophysin (SYN), glutamic acid decarboxylase 65/67 (GAD6), or vesicular glutamate transporter 1 (VGluT1) in any of the regions studied (Figure 3 and Additional File 2, Table S1)). We did not find changes in the density of puncta colocalizing PSA-NCAM/SYN, PSA-NCAM/GAD6 or PSA-NCAM/VGluT1 neither in the PrL nor in the Cg2 (Figure 2 and Additional File 2, Table S1).

We did not find changes neither in the dot size nor in the surface covered by puncta in any of the regions and the markers analyzed (Additional File 3, Figure S2).

#### Amygdala

As described previously [24], low levels of PSA-NCAM expression were found in the neuropil of the lateral and basolateral amygdaloid nuclei. By contrast, PSA-NCAM expression was denser and more intense in the central, medial and basomedial nuclei (Figure 2).

**Figure 4:**
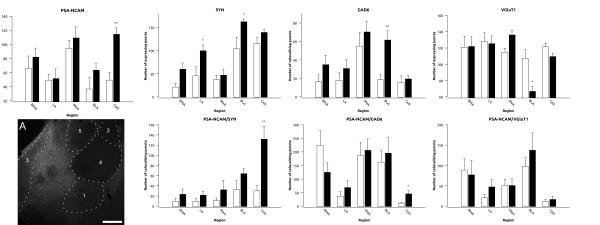
**Graphs representing the expression of puncta for the different markers and its colocalization in different areas of the amygdala**. White bars represent control animals and black bars represent fluoxetine treated animals respectively. A is a focal plane showing the expression of PSA-NCAM in the (1) basomedial (2) lateral, (3) medial, (4) basolateral and (5) central nuclei of the amygdala. Scale bar: 200 μm.

In the fluoxetine treated group we found significant increases in the density of PSA-NCAM expressing puncta in the central amygdaloid nucleus (p = 0.001), of SYN expressing puncta in the lateral (p = 0.049) and basolateral amygdaloid nuclei (p = 0.047) and of GAD6 expressing puncta in the basolateral nucleus (p = 0.005) (Figure 4 and Additional File 2, Table S1). On the contrary, we found a significant decrease in the number of VGluT1 expressing puncta in the basolateral amygdaloid nucleus (p = 0.04) (Figure 4 and Additional File 2, Table S1). We also found an increase in PSA-NCAM/SYN (p = 0.007) and PSA-NCAM/GAD6 (p = 0.041) expressing puncta in the central amygdala. No changes in the density of these double-labeled puncta were observed in any of the other amygdaloid nuclei studied (Figure 4 and Additional File 2, Table S1).

We also found changes similar to those described for puncta density when analyzing the dot size and the surface covered by these puncta. In the central amygdaloid nucleus we observed an increase both in the dot size and the surface covered by PSA-NCAM expressing puncta (p = 0.027 and 0.011). In the lateral nucleus we only observed an increase in the dot size of puncta expressing SYN (p = 0.038). By contrast, in the basolateral nucleus we found an increase in the dot size and surface covered by puncta expressing GAD6 (p = 0.011 and 0.018) and a decrease in both, the dot size and the surface covered by puncta expressing VGluT1 (p = 0.005 and 0.007) (Additional File 4, Figure S3).

#### Hippocampus

PSA-NCAM expression in the hippocampus was similar to that described previously [26]: briefly, the most intense expression of PSA-NCAM was located in the somata and apical dendrites of granule cells in the SGZ of the dentate gyrus, as well as in the neuropil of the hilus and the mossy fibers. A weaker and more diffuse expression of PSA-NCAM could be found in the neuropil of other regions of the hippocampus, such as the molecular layer of the dentate gyrus and the strata lacunosum-moleculare, radiatum and oriens of CA1 and CA3 (Figure 2), where PSA-NCAM expressing cell somata could also be found [29].

**Figure 5:**
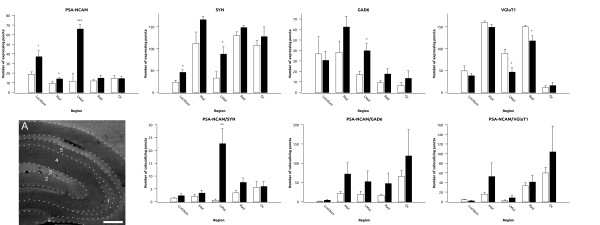
**Graphs representing the expression of puncta for the different markers and its colocalization in different areas of the hippocampus**. White bars represent control animals and black bars represent fluoxetine treated animals respectively. A is a focal plane showing the expression of PSA-NCAM in the strata (1) Lucidum, (2) Molecular, (3) Lacunosum Moleculare, (4) Radiatum and (5) Oriens of the hippocampus. Scale bar: 200 μm.

After chronic fluoxetine treatment, we found significant increases in the density of PSA-NCAM expressing puncta in the molecular layer (p = 0.045), stratum lucidum (p = 0.032) and stratum lacunosum-moleculare (p < 0.001) (Figure 5 and Additional File 2, Table S1). Similar increases were found in the density of SYN expressing puncta in the strata lucidum (p = 0.012) and lacunosum-moleculare (p = 0.038). We also found a significant increase (p = 0.018) in the density of GAD6 expressing puncta in the stratum lacunosum-moleculare. By contrast, we found a decrease in the density of VGluT1 expressing puncta in the strata lacunosum-moleculare (p = 0.021) and radiatum (p = 0.030) of CA1 (Figure 5 and Additional File 2, Table S1). When studying the density of puncta co-expressing PSA-NCAM and each of the other markers, we observed an increase in the density of puncta co-expressing PSA-NCAM/SYN (p = 0.008) in the stratum lacunosum-moleculare, but no change was observed in those co-expressing PSA-NCAM/GAD or PSA-NCAM/VGluT1 (Figure 5 and Additional File 2, Table S1).

Finally, when analyzing the dot size and the surface covered by immunoreactive puncta, we also observed changes in most of the regions that showed differences in the density of puncta in these structures. In the stratum lacunosum-moleculare we observed an increase in the dot size and the surface covered by puncta expressing PSA-NCAM (p = 0.011 and < 0.001) and GAD6 (p = 0.047 and 0.021), while there was only an increase in dot size in those expressing SYN (p = 0.044) and a decrease in both dot size and surface covered by those expressing VGluT1 (p = 0.035 and 0.017). We also find a decrease in both the dot size and the surface covered by puncta expressing VGluT1 in the stratum radiatum (p = 0.015 and 0.007) (Additional File 5, Figure S4).

### Adult neurogenesis

**Figure 6:**
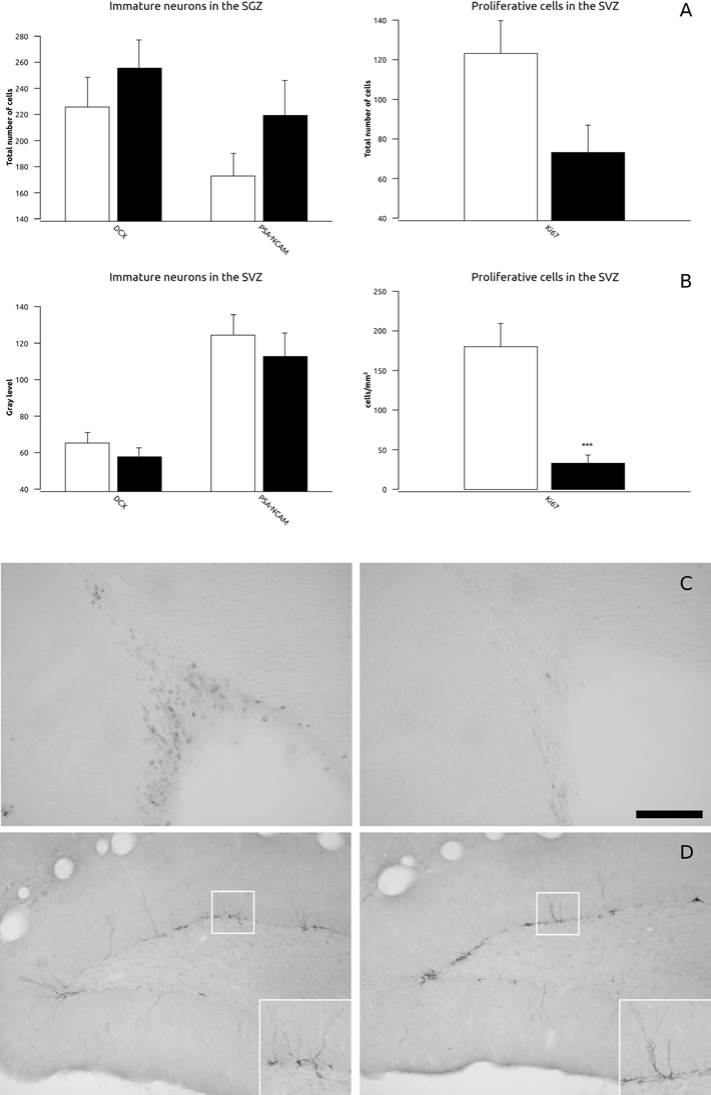
**Graphs representing (A) the total number of cells expressing immature and proliferative markers in the subgranular zone (SGZ) of the hippocampus and (B) the gray level of doublecortin (DCX) and PSA-NCAM and the density of Ki67 in the subventricular zone (SVZ)**. White bars represent control animals and black bars represent fluoxetine treated animals respectively. (C) Micrographs showing the expression of Ki67 in the SVZ and (D) DCX in the SGZ. Scale bar: 100 μm (C and D) and 50 μm (insets in D).

No change in the density of cells expressing DCX or PSA-NCAM was observed in the subgranular zone (SGZ) of the dentate gyrus after chronic fluoxetine treatment (Figure 6). However, when analyzing the number of Ki67 expressing nuclei there was a trend towards a decrease in the treated group (p = 0.061) (Figure 6). In the subventricular zone (SVZ) we also failed to find changes in the expression of DCX or PSA-NCAM, but we observed a dramatic significant decrease in the number of Ki67 immunoreactive nuclei in the treated group (p < 0.001) (Figure 6).

## Discussion

Our results indicate that in middle-aged rats chronic fluoxetine treatment induces changes in the expression of molecules related to neuronal structural plasticity. These changes occur in the same direction of those observed previously in young-adult animals in the hippocampus and the mPFC, although to a reduced extent in this later region. However, they follow the opposite direction in the amygdala. We have also observed that, in contrast with what has been observed in young-adult rats, there is a negative impact of fluoxetine **on cell proliferation **in the SVZ and apparently a lack of effect in the SGZ.

### PSA-NCAM expression as an indicator of neuronal structural plasticity

Previous experiments have demonstrated that PSA-NCAM expression is sensible to fluoxetine treatment, acting through 5HT3 receptors [20]. However, since other 5HT receptors are known to mediate the effects of fluoxetine, they may also influence PSA-NCAM expression [46-51]. Many PSA-NCAM expressing structures in the cerebral cortex [24,29,30] and the amygdala [52] of rodents belong to mature interneurons. Consequently, changes in PSA-NCAM expression should primarily affect the structure of these inhibitory. In this line, we have recently reported that PSA-NCAM expressing cortical interneurons have reduced synaptic input and decreased dendritic arborization and spine density when compared with neighboring interneurons lacking PSA-NCAM [30]. In fact, a previous report from our laboratory using a dopamine D2 receptor antagonist, which increases PSA-NCAM expression in the mPFC, also resulted in a parallel upregulation of GAD67 expression [53]. It is possible then, that the changes in PSA-NCAM expression observed in the present study affect the connectivity of certain interneurons, regulating the surface of preexisting plasma membrane available for the establishment of synaptic contacts. Another non-excluding possibility may be that, given its anti-adhesive properties, changes in PSA-NCAM expression may regulate the ability of certain interneurons to remodel the structure of their neurites, and consequently their connections, in response to fluoxetine treatment.

### Comparison of the effects of chronic fluoxetine treatment on the expression of PSA-NCAM and presynaptic markers between middle-aged and young-adult animals

The present results in the mPFC, showing only an increase in the density of PSA-NCAM expressing puncta in the dorsal cingulate cortex, are more restricted than those found previously in young-adult rats, which showed increases in every region and layer of the mPFC [20]. The lack of changes in the density of puncta expressing presynaptic markers is also in contrast with previous reports using young-adult rats, which showed increases in SYN expression in deep layers of the prelimbic and infralimbic cortices [21] and of VGluT-1 mRNA expression in the cingulate cortex [54].

In the hippocampus, previous studies with young-adult rats found a similar increase in PSA-NCAM expression in the stratum lucidum, but not in the rest of hippocampal strata studied [21]. Since in young-adult rats antidepressants promote an increase in the density of PSA-NCAM expressing cells in the SGZ [17], fluoxetine may be affecting PSA-NCAM expression in the stratum lucidum by increasing the production or accelerating the maturation of immature granule cells, some of which may have already sent PSA-NCAM expressing mossy fibers to this layer [55]. However, our results clearly indicate that the number of these cells is not affected by fluoxetine in middle-aged rats, suggesting that changes in PSA-NCAM expressing puncta are not linked to adult neurogenesis. In connection to this, a recent report has shown that chronic fluoxetine treatment may produce a dematuration of granule cells [56]. Since many mossy fibers in the stratum lucidum are polysialylated by St8SiaII, the enzyme responsible for the polysialylation of immature neurons in the adult cerebral cortex [57], this putative dematuration induced by fluoxetine may explain the increase in PSA-NCAM expression that we observe in this stratum. In consonance with this hypothesis and with previous studies in young-adult rats [21,58], we have failed to find any significant difference in the density of SYN expressing puncta in the stratum lucidum, which may indicate that the changes in PSA-NCAM are not related to the genesis of new functional synaptic contacts. By contrast, the increase of PSA-NCAM expressing puncta in the stratum lacunosum-moleculare of middle-aged rats is accompanied by an increase in the density of SYN expressing puncta and of puncta co-expressing PSA-NCAM/SYN, suggesting that these structures correspond to synapses. Interestingly, in the stratum lacunosum-moleculare of middle-aged animals we also found an increase in the density of GAD6 expressing puncta, and a decrease in the density of those expressing VGluT1, which may indicate that the upregulation of PSA-NCAM expression may be linked to changes in inhibitory circuits, probably involving the formation of inhibitory synapses.

Chronic fluoxetine treatment in young-adult rats decreases PSA-NCAM and SYN expression in most amygdaloid nuclei [21] and decreases the number of PSA-NCAM expressing neurons in this limbic region [59]. By contrast, in middle-aged rats we not only have failed to observe decreases in the density of puncta expressing PSA-NCAM or SYN, but have observed significant increases in the density of PSA-NCAM expressing puncta in the central nucleus and of those expressing SYN in the lateral and basolateral nuclei. There is, in fact, a general tendency for an increase in the density of puncta expressing PSA-NCAM, SYN and GAD6 in all the amygdaloid nuclei studied. Moreover, despite the fact that no significant changes in puncta expressing exclusively GAD6 or SYN were observed in the central amygdala, the density of puncta coexpressing PSA-NCAM and each of these two markers also increases significantly, suggesting that changes in PSA-NCAM expression may be associated to the generation or remodeling of inhibitory synapses in this region. In the basolateral amygdala, chronic fluoxetine treatment may also influence positively synaptic plasticity of inhibitory circuits, as indicated by increased densities of GAD6 and SYN expressing puncta, while the density of those expressing VGluT1 is decreased.

It is possible that substantial differences in 5HT content and in the expression of its receptors, transporters, etc... exist in middle-aged animals. The levels of 5HT are generally reduced in the brain of aged rats [60] and, although there are no studies in middle-aged animals, it is possible that they are already reduced at this age. There is also evidence indicating that the expression 5HT1A receptor, which has an important role in the antidepressant effects of 5HT reuptake inhibitors [61,62], is lower in young-adult than in adolescent rats [59]. Additionally, other factors such as changes in inhibitory neurotransmission may also influence the effects of fluoxetine in middle-aged animals, particularly in the amygdala, where decreases in GABA concentration and hyperactivity have been described during aging [63,64].

### Effects of fluoxetine treatment on neurogenesis

The present results on adult hippocampal neurogenesis are in agreement with previous studies showing that neither proliferation nor neuronal survival/differentiation is affected by chronic antidepressant treatment in middle-aged rodents [32,37]. This lack of response of the SGZ in middle-aged animals must be due to changes that alter the molecular pathways by which 5HT stimulates neurogenesis. There is an age-dependent reduction in the levels of 5HT [60] and some of its receptors [65] in the hippocampus. Since glucocorticoids are important modulators of adult hippocampal neurogenesis and these steroids are particularly elevated during aging [66], they may also influence the neurogenic response to antidepressants in middle-aged animals. In fact, glucocorticoids decrease 5HT transporter expression [67] and the neurogenic action of fluoxetine is blocked by a flattened corticosterone rhythm induced by artificially enhanced glucocorticoid levels [68]. Additionally, a previous study has clearly shown that intraperitoneal injections *per se *have effects on the structure of neurons, at least in the mPFC [69]. This effects are similar to those observed after chronic stress [41]and it is very likely that this procedure also affects some of the plasticity parameters measured in our study, including neurogenesis, which is importantly influenced by chronic stress [8].

Other molecules known to modulate adult neurogenesis, including neurotransmitters, such as glutamate acting on NMDA receptors [70] and GABA [71], or trophic factors, such as BDNF [72], can be influenced by antidepressants, including fluoxetine [73-76]. Consequently, age-dependent changes in their expression or in the molecular pathways in which these molecules are involved may also lead to changes in the pro-neurogenic effects of antidepressants.

In contrast with previous studies using fluoxetine [31] and 5HT receptor agonists [77] in young-adult rodents, which found increases in the number of proliferating cells in the SVZ, we have found the opposite effect. On the other hand, our results are in agreement with a recent report describing that in young-adult mice 3 weeks of fluoxetine treatment do not affect cell proliferation in the SVZ, but that the extension of this treatment to 6 weeks produces a significant deficit in the number of dividing cells in this region [78]. These results indicate that the SVZ of middle-aged rodents is more sensible to fluoxetine treatment, since a significant decrease in cell proliferation can be achieved with a shorter treatment. It is possible that the lack of differences in the number of neuroblasts in the SVZ may be due to a homeostatic parallel change in the number of apoptotic cells in this region, as suggested previously [79]. Alternatively, fluoxetine could impair neuronal migration, which may cause accumulation of neuroblasts in the SVZ.

### Implication of the present results on antidepressant therapy

In light of the neuroplastic hypothesis of depression [1], our results indicate that antidepressant treatment in middle-aged individuals also has an important impact on neuronal structure and connectivity. Consequently, it may have relevant consequences on the therapeutics of major depression. In the present results, we have shown that chronic fluoxetine treatment increases the volume of the hippocampus and the expression of molecules related to structural plasticity and inhibitory neurotransmission. These phenomena may counteract the decreases in hippocampal volume and balance the increases in excitatory neurotransmission observed in depressed patients and in animal models of this disorder [8]. Similar, although lighter, effects have been observed in the amygdala. In this region, the increases in the expression of molecules related to structural plasticity and inhibitory neurotransmission may compensate the structural changes observed in inhibitory and excitatory neurons, which accompany the increased excitation observed in this limbic region in animal models of depression [80]. The effects of fluoxetine in the mPFC of middle-aged rats are more discrete, but it is reasonable to think that the increase in PSA-NCAM expression may facilitate compensatory structural changes to overcome the structural atrophy of pyramidal neurons observed in animal models of depression [42]**.**

By contrast, our results on the effects of fluoxetine on adult neurogenesis do not seem to give support to the idea that this type of plasticity is important for the treatment of depression, at least in middle-aged rodents. It has to be noted, however, that our experiment has been performed in control animals and that the effects of fluoxetine on molecules related to neuronal structural plasticity or in neurogenesis may be more intense or follow a different direction when applied to animal models of this psychiatric disorder.

## Conclusions

Our results indicating substantial differences in the plastic response of the CNS to fluoxetine in middle-aged animals may be relevant when considering what are the cellular bases of the behavioral changes associated to antidepressant action.

As it happens with rodents, middle-aged humans may also show reduced plasticity in response to antidepressant and, what is more important, this plasticity may follow a different direction in certain brain regions, such as the SVZ or the amygdala. These results indicate that the response to antidepressant treatment is more complex than previously thought and that the age of the subjects receiving these drugs is an important factor to consider and a subject for more intensive research.

## Competing interests

The authors declare that they have no competing interests.

## Authors' contributions

RG carried out the animal experimentation, the histology, the image acquisition and processing, the statistics and drafted the manuscript. DSM participated in the image acquisition and processing. EV, CC and JMBI have revised critically the content of the manuscript. JN conceived and designed the experimental work, helped to draft the manuscript and wrote its final version. All authors read and approved the final manuscript.

## Supplementary Material

Additional file 1 **Quantification of PSA-NCAM and SYN expressing puncta in the hippocampus**. Confocal image showing the expression of PSA-NCAM and SYN in the hippocampus. Focal planes showing the original image and the processed image for the puncta analysis for (B1 and B2) PSA-NCAM, (C1 and C2) SYN and (D1 and D2) the composite image. Scale bar: 320 μm in A and 5 μm in the rest. Click here for file

Additional file 2 **Statistical values of puncta expressing different markers**. Statistical information of the expression of the different markers and its colocalization in the diferent regions of the mPFC, the hippocampus and the amygdala. Click here for file

Additional file 3 **Graphs for the dot size and surface covered by puncta expressing different markers in the Mpfc**. Graphs representing the dot size and surface covered by puncta expressing different markers in the prelimbic and cingulate cortex of the medial prefrontal cortex. White bars represent control animals and black bars represent fluoxetine treated animals respectively. Click here for file

Additional file 4 **Graphs for the dot size and surface covered by puncta expressing different markers in the amygdala**. Graphs representing the dot size and surface covered by puncta expressing different markers in different areas of the amygdala. White bars represent control animals and black bars represent fluoxetine treated animals respectively. Click here for file

Additional file 5 **Graphs for the dot size and surface covered by puncta expressing different markers in the hippocampus**. Graphs representing the dot size and surface covered by puncta expressing different markers in different areas of the hippocampus. White bars represent control animals and black bars represent fluoxetine treated animals respectively. Click here for file
