# Supplementary figures and images for: Chronic fluoxetine treatment in middle-aged rats induces changes in the expression of plasticity-related molecules and in neurogenesis
Source: BMC Neurosci. 2012 Jan 5;13:5. doi: 10.1186/1471-2202-13-5 (PMC3278353; doi:10.1186/1471-2202-13-5)

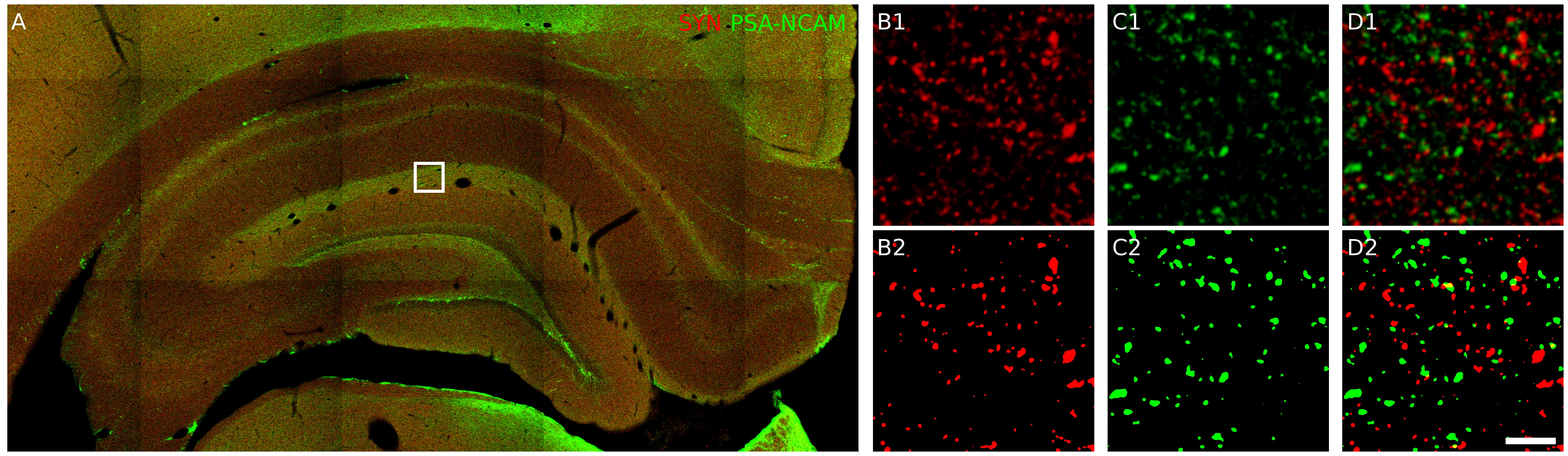

Supplement: Additional file 1 — Quantification of PSA-NCAM and SYN expressing puncta in the hippocampus. Confocal image showing the expression of PSA-NCAM and SYN in the hippocampus. Focal planes showing the original image and the processed image for the puncta analysis for (B1 and B2) PSA-NCAM, (C1 and C2) SYN and (D1 and D2) the composite image. Scale bar: 320 μm in A and 5 μm in the rest. [file 1471-2202-13-5-S1.JPEG]

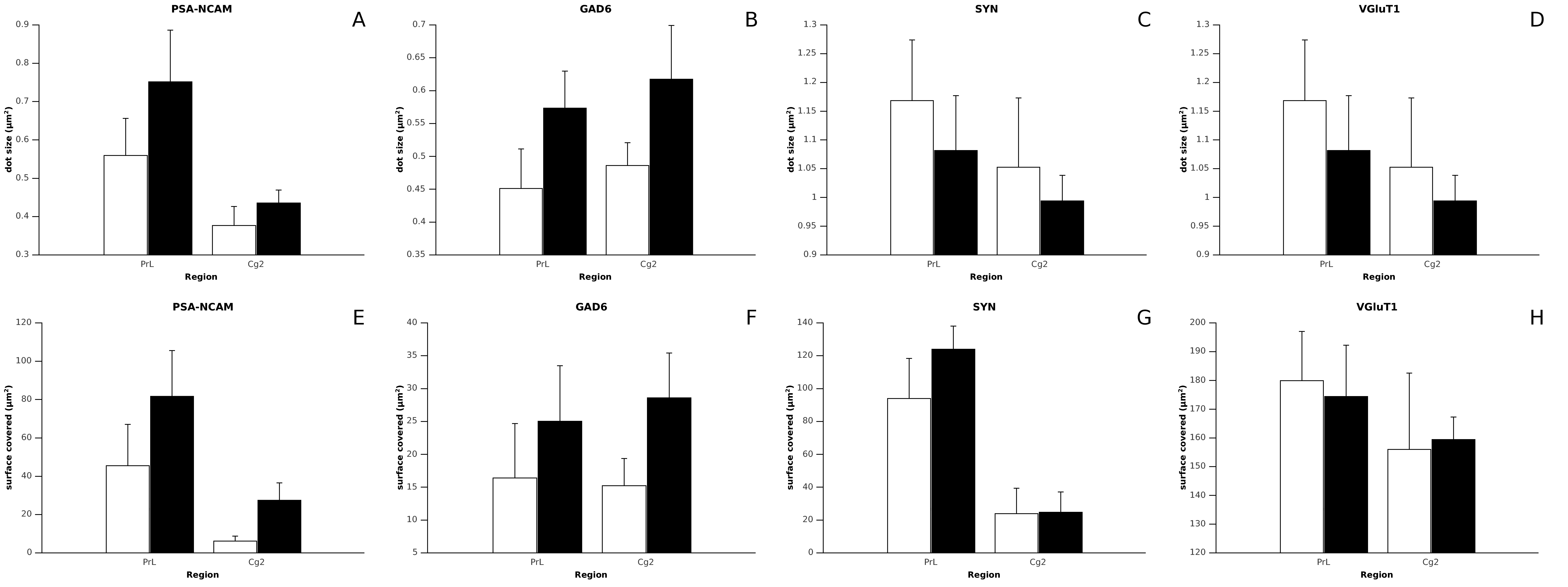

Supplement: Additional file 3 — Graphs for the dot size and surface covered by puncta expressing different markers in the Mpfc. Graphs representing the dot size and surface covered by puncta expressing different markers in the prelimbic and cingulate cortex of the medial prefrontal cortex. White bars represent control animals and black bars represent fluoxetine treated animals respectively. [file 1471-2202-13-5-S3.JPEG]

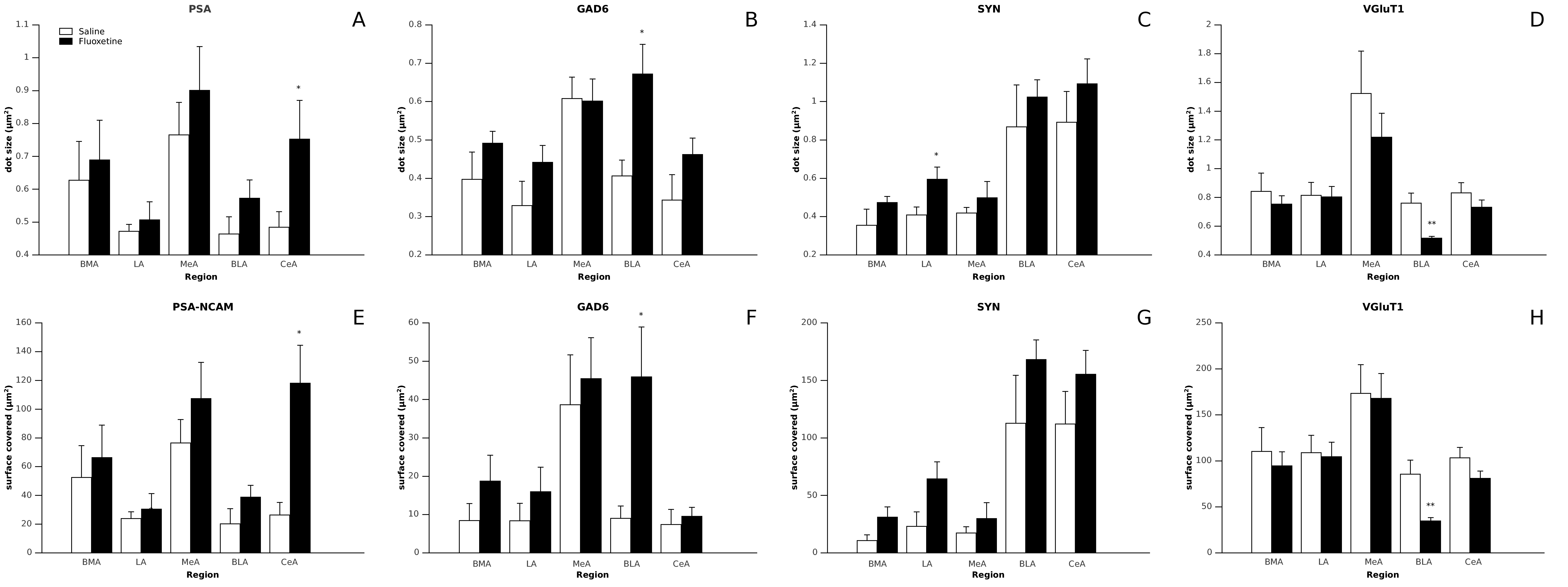

Supplement: Additional file 4 — Graphs for the dot size and surface covered by puncta expressing different markers in the amygdala. Graphs representing the dot size and surface covered by puncta expressing different markers in different areas of the amygdala. White bars represent control animals and black bars represent fluoxetine treated animals respectively. [file 1471-2202-13-5-S4.JPEG]

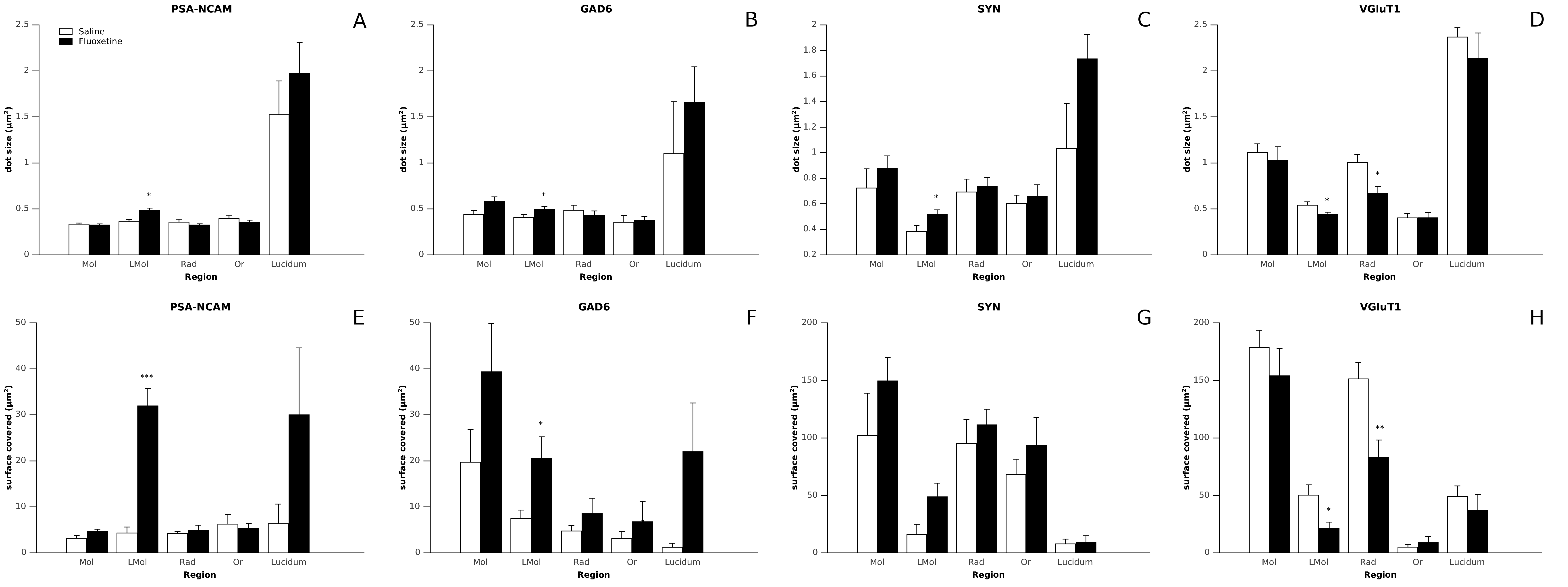

Supplement: Additional file 5 — Graphs for the dot size and surface covered by puncta expressing different markers in the hippocampus. Graphs representing the dot size and surface covered by puncta expressing different markers in different areas of the hippocampus. White bars represent control animals and black bars represent fluoxetine treated animals respectively. [file 1471-2202-13-5-S5.JPEG]
