# Supplementary material for: Chronic fluoxetine treatment in middle-aged rats induces changes in the expression of plasticity-related molecules and in neurogenesis
Source: BMC Neurosci. 2012 Jan 5;13:5. doi: 10.1186/1471-2202-13-5 (PMC3278353; doi:10.1186/1471-2202-13-5)
Supplement: Additional file 2 — Statistical values of puncta expressing different markers. Statistical information of the expression of the different markers and its colocalization in the diferent regions of the mPFC, the hippocampus and the amygdala. [file 1471-2202-13-5-S2.PDF]

| Marker | Area | Region | Saline mean | Treated mean | p-value |
|---|---|---|---|---|---|
| PSA-NCAM | mPFC | PrL | 64.6±18.8 | 99.3±13.3 | 0.197 |
| | | Cg2 | 13.5±6.6 | 50.3±13.2 | 0.043 |
| | Hippocampus | Mol | 9.5±1.8 | 14.2±1.2 | 0.045 |
| | | LMol | 11.6±8.2 | 65.7±4.4 | <0.001 |
| | | Rad | 12±1.3 | 15±2.8 | 0.418 |
| | | Or | 14.6±3.1 | 14.6±1.9 | 0.982 |
| | | Luc | 19±2.36 | 37.1±6.1 | 0.032 |
| | Amygdala | BMA | 65.8±17.7 | 82±12.7 | 0.512 |
| | | LA | 49.5±7.8 | 51.7±13.7 | 0.899 |
| | | MeA | 94.5±11 | 109±15.6 | 0.494 |
| | | BLA | 37±16 | 63.6±8.9 | 0.190 |
| | | CeA | 49.5±11 | 114.1±8.9 | 0.001 |
| SYN | mPFC | PrL | 133±12.8 | 161.7±7.78 | 0.101 |
| | | Cg2 | 41.5±19.8 | 47.14±18.2 | 0.856 |
| | Hippocampus | Mol | 112.3±26 | 166.6±7.3 | 0.068 |
| | | LMol | 33.2±14.5 | 88±16.3 | 0.038 |
| | | Rad | 130.2±8.5 | 148.7±4.7 | 0.103 |
| | | Or | 108.2±10.8 | 127.9±22 | 0.491 |
| | | Luc | 23.8±4.5 | 46.29±5.8 | 0.012 |
| | Amygdala | BMA | 21.8±8.2 | 60.6±13.2 | 0.050 |
| | | LA | 46±19.6 | 100.3±12.5 | 0.049 |
| | | MeA | 38±9.6 | 47.6±12.1 | 0.588 |
| | | BLA | 104.6±24.9 | 162.9±5.9 | 0.047 |
| | | CeA | 116±13.1 | 139.9±6.1 | 0.141 |
| GAD6 | mPFC | PrL | 27.6±10.6 | 38.9±8.5 | 0.458 |
| | | Cg2 | 29.1±6.7 | 42.1±6.3 | 0.217 |
| | Hippocampus | Mol | 38.4±10.6 | 62.4±10.2 | 0.157 |
| | | LMol | 17±3.4 | 39.9±6.9 | 0.018 |
| | | Rad | 9.3±1.9 | 17.7±5.2 | 0.187 |
| | | Or | 6.9±2.5 | 13.6±7.2 | 0.434 |
| | | Luc | 36.8±16.3 | 30.7±8.7 | 0.736 |
| | Amygdala | BMA | 16.6±7.8 | 35.1±10.2 | 0.205 |
| | | LA | 18.1±8.3 | 31±9.5 | 0.363 |
| | | MeA | 55.1±14.9 | 70.4±11.2 | 0.462 |
| | | BLA | 18.7±5.5 | 61.7±10.2 | 0.005 |
| | | CeA | 16±6.9 | 19.7±3.6 | 0.669 |
| VGluT1 | mPFC | PrL | 153.4±2.4 | 159.7±3.6 | 0.204 |
| | | Cg2 | 138.4±17.5 | 160.6±3.9 | 0.275 |
| | Hippocampus | Mol | 160.3±3.3 | 149.3±6.3 | 0.180 |
| | | LMol | 89.6±9.7 | 46.9±11.2 | 0.021 |
| | | Rad | 150.4±3 | 118.1±11.8 | 0.030 |
| | | Or | 11.1±4.3 | 16.1±7.4 | 0.600 |
| | | Luc | 49.5±11.4 | 45.8±5.1 | 0.765 |
| | Amygdala | BMA | 121±13.5 | 121.9±12.1 | 0.902 |

| | | | | | |
|---|---|---|---|---|---|
| | | LA | 128±10.7 | 125.3±9.3 | 0.862 |
| | | MeA | 114.9±4.7 | 135.9±5.6 | 0.050 |
| | | BLA | 107.3±10.4 | 67.1±5.9 | 0.042 |
| | | CeA | 121.4±4.6 | 109.6±4 | 0.059 |
| PSA-NCAM/SYN | mPFC | PrL | 29.8±10.9 | 80.3±20.5 | 0.083 |
| | | Cg2 | 3.2±1.8 | 12.7±6.9 | 0.270 |
| | Hippocampus | Mol | 2.2±0.8 | 3.4±1.1 | 0.430 |
| | | LMol | 0.7±0.4 | 22.6±5.9 | 0.008 |
| | | Rad | 3.5±1.02 | 7.6±1.7 | 0.100 |
| | | Or | 5.6±2.2 | 6±1.92 | 0.914 |
| | | Luc | 1.5±0.2 | 2.4±0.8 | 0.311 |
| | Amygdala | BMA | 10.5±5.5 | 23.9±10.4 | 0.34 |
| | | LA | 10.2±5.1 | 22.3±7.7 | 0.26 |
| | | MeA | 12±4.7 | 32.7±18.2 | 0.362 |
| | | BLA | 32.8±18.2 | 64.1±10 | 0.174 |
| | | CeA | 30.5±10 | 131.4±25.1 | 0.007 |
| PSA-NCAM/GAD6 | mPFC | PrL | 62.6±36.9 | 39±10.1 | 0.551 |
| | | Cg2 | 86.6±16.7 | 81.8±29.6 | 0.901 |
| | Hippocampus | Mol | 21.6±5.5 | 72.1±29.1 | 0.173 |
| | | LMol | 20±7.4 | 52.4±27.4 | 0.339 |
| | | Rad | 17±2.2 | 47.6±27 | 0.372 |
| | | Or | 66.3±15 | 103.4 | 0.526 |
| | | Luc | 2±0.3 | 4.5±2 | 0.308 |
| | Amygdala | BMA | 223.3±53.9 | 125.1±35.6 | 0.173 |
| | | LA | 38.2±16.9 | 69.6±25.5 | 0.379 |
| | | MeA | 187±48.5 | 205.6±41.9 | 0.790 |
| | | BLA | 162±41.9 | 195.1±58 | 0.68 |
| | | CeA | 12.6±2.9 | 46.4±10.9 | 0.041 |
| PSA-NCAM/VGluT1 | mPFC | PrL | 48.8±26.2 | 13.1±4 | 0.204 |
| | | Cg2 | 46±8.1 | 48.1±16 | 0.908 |
| | Hippocampus | Mol | 16.1±3.1 | 52.6±28.1 | 0.293 |
| | | LMol | 2.8±1.2 | 8.6±5.4 | 0.387 |
| | | Rad | 33.2±6.9 | 41.1±14.2 | 0.660 |
| | | Or | 59.8±11.7 | 103.4±53.2 | 0.503 |
| | | Luc | 4.3±1.2 | 2.3±0.8 | 0.186 |
| | Amygdala | BMA | 88.8±27.4 | 77.3±35 | 0.821 |
| | | LA | 21.3±8.2 | 47.9±18.5 | 0.274 |
| | | MeA | 50.7±15.7 | 51.1±16.2 | 0.982 |
| | | BLA | 97.3±22.4 | 137.3±42.2 | 0.475 |
| | | CeA | 12.5±5.2 | 17.3±6.9 | 0.621 |
